# Identification of small RNAs in *Francisella tularensis*

**DOI:** 10.1186/1471-2164-11-625

**Published:** 2010-11-10

**Authors:** Guillaume Postic, Eric Frapy, Marion Dupuis, Iharilalao Dubail, Jonathan Livny, Alain Charbit, Karin L Meibom

**Affiliations:** 1 INSERM U1002, Paris, France; 2 Université Paris Descartes, Faculté de Médecine Necker-Enfants Malades, Paris, France; 3 Broad Institute of MIT and Harvard, Cambridge, USA; 4 Channing Laboratories, Brigham and Women's Hospital, Harvard Medical School, Boston, USA

## Abstract

**Background:**

Regulation of bacterial gene expression by small RNAs (sRNAs) have proved to be important for many biological processes. *Francisella tularensis *is a highly pathogenic Gram-negative bacterium that causes the disease tularaemia in humans and animals. Relatively little is known about the regulatory networks existing in this organism that allows it to survive in a wide array of environments and no sRNA regulators have been identified so far.

**Results:**

We have used a combination of experimental assays and *in silico *prediction to identify sRNAs in *F. tularensis *strain LVS. Using a cDNA cloning and sequencing approach we have shown that *F. tularensis *expresses homologues of several sRNAs that are well-conserved among diverse bacteria. We have also discovered two abundant putative sRNAs that share no sequence similarity or conserved genomic context with any previously annotated regulatory transcripts. Deletion of either of these two loci led to significant changes in the expression of several mRNAs that likely include the cognate target(s) of these sRNAs. Deletion of these sRNAs did not, however, significantly alter *F. tularensis *growth under various stress conditions *in vitro*, its replication in murine cells, or its ability to induce disease in a mouse model of *F. tularensis *infection. We also conducted a genome-wide *in silico *search for intergenic loci that suggests *F. tularensis *encodes several other sRNAs in addition to the sRNAs found in our experimental screen.

**Conclusion:**

Our findings suggest that *F. tularensis *encodes a significant number of non-coding regulatory RNAs, including members of well conserved families of structural and housekeeping RNAs and other poorly conserved transcripts that may have evolved more recently to help *F. tularensis *deal with the unique and diverse set of environments with which it must contend.

## Background

RNA regulators are important players in control of gene expression in bacteria and often mediate a response to changes in the environment (for review, see [1]). Some regulatory RNAs, designated riboswitches, are part of the mRNA they regulate. Riboswitches are sequences in the 5' end of mRNAs that change conformation upon binding of a ligand, affecting transcription or translation of the down-stream gene (positively or negatively). Other RNA regulators bind to proteins and regulate their function, whereas the largest group of small RNAs (sRNAs), act by base pairing with target RNAs. Base pairing sRNAs generally fall into two groups: *cis*-acting sRNAs that have capacity for extensive base pairing, and *trans*-encoded sRNAs with a more limited potential for base pairing with their target RNA. *Trans*-acting sRNAs regulate the translation and/or the stability of their target RNAs and each often regulate more than one target. For the most part, these sRNAs affect target genes in a negative fashion by binding to the region surrounding the start codon and ribosome binding site, but can act through base pairing in a region far upstream, and occasionally affect translation positively (for review see [1]). Many of the *trans*-encoded sRNAs require the RNA chaperone Hfq for function. Hfq promotes RNA-RNA interactions between the sRNA and its target mRNA and the protein may additionally stabilize the sRNA *in vivo*.

RNA regulators furthermore control pathogenesis in bacteria such as *Staphylococcus aureus *[2-6], *Salmonella typhimurium *[7-10], *Vibrio cholerae *[11-13], group A *Streptococcus *[14,15], *Pseudomonas aeruginosa *[16-19], *Clamydia trachomatis *[20], and *Clostridium perfringens *[21,22].

*Francisella tularensis *is a Gram-negative bacterium that causes the disease tularaemia in humans and in a large number of animals and is one of the most virulent bacterial pathogens known. The ability of *F. tularensis *to invade and replicate in host cells, particularly in immune cells such as macrophages, is critical for its capacity to cause disease. Once inside host cells, *F. tularensis *resides transiently inside a phagosome that matures into a late endosomal stage and the bacterium then escapes to replicate in the cytoplasm. The mechanism by which the bacterium escapes the phagosome is not well understood, but genes encoded in a pathogenicity island (FPI) are required for this step in the infectious cycle. Several regulatory proteins regulate virulence gene expression in *F. tularensis *by activating transcription of the FPI. These comprise the proteins MglA [23,24], SspA [25], PrmA [26], and FevR (or PigR) [27,28]. *F. tularensis *unlike many other bacteria encodes only one alternative sigma factor [29], no complete two component regulator pairs [26], and very little information exists concerning control of gene expression apart from the above mentioned regulation of the FPI.

We have recently studied the role of Hfq in the physiology and virulence of *F. tularensis *[30]. Transcriptional analyses revealed that Hfq - directly or indirectly - regulates the expression of numerous genes in this pathogen. Functional studies showed that Hfq is required for stress resistance as well as for full virulence in both a fully virulent strain and in the attenuated live vaccine strain (LVS). Since Hfq normally performs its function by promoting sRNA-mRNA interactions, these results strongly suggest that sRNAs are expressed in this organism and are involved in diverse functions. Here, we have initiated the identification of the sRNAs expressed by *F. tularensis *LVS. We have assessed and identified the sRNA species that are expressed at high levels and found the commonly known 4.5S RNA, 5S rRNA, and the transfer messenger RNA (tmRNA). Additionally, we identified two novel sRNA species by cDNA cloning and have characterized these. Studies of sRNA mutant strains suggested potential targets for regulation. As an alternative approach to identify sRNAs in *F. tularensis*, we performed a bioinformatic prediction of sRNAs. Interestingly, this analysis found both the experimentally determined sRNA loci and additionally identified a number of other putative sRNA-containing intergenic regions.

## Results and Discussion

### Identification of highly expressed small RNA species of *Francisella*

**Figure 1:**
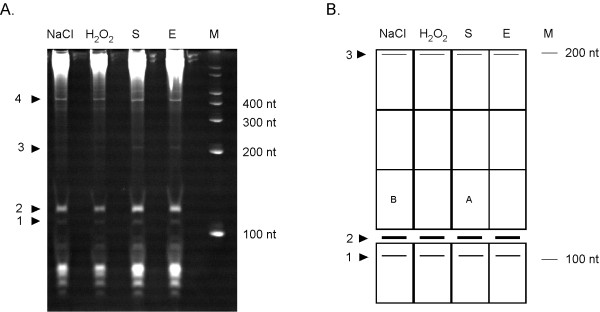
**Experimental identification of *F. tularensis *sRNAs**. **(A) **Total RNA extracted at exponential phase (E), stationary phase (S), high salt concentration (NaCl) or after exposure to oxidative stress (H_2_O_2_) analyzed by 8% PAGE and stained by ethidium bromide (M, RNA marker). Individual bands processed for cloning are indicated by numbers. **(B) **Schematic representation of gel used for cloning of cDNA. Each square denotes a piece that was individually processed for RNA extraction and A and B designate the area from which FtrA and B were identified, respectively. For clarification, the highly expressed RNAs (#1-3) seen in (A) are indicated.

To identify sRNAs in *F. tularensis *we first assessed the highly expressed sRNAs by polyacrylamide gel electrophoresis followed by ethidium bromide staining (Figure 1). This approach has been used successfully to find novel sRNAs in *S. aureus *[31]. We isolated total RNA from *F. tularensis *LVS after growth in normal complex broth in exponential growth phase (E) or in stationary phase (S), after oxidative stress (10 mM H_2_O_2_) or after growth at high osmolarity (Schaedler broth + 2% NaCl). We chose to assess the RNA profile under different conditions because sRNA expression in other bacteria was found to be constitutive for some sRNAs (mostly *cis*-encoded sRNAs), but induced under specific conditions (such as exposure to stress) for most *trans*-encoded sRNAs [1]. However, no major differences were observed in the pattern of RNA species that were visible after PAGE (Figure 1A). Four bands ranging in size from ~100 nt to ~400 nt were visible after staining (Figure 1A; labeled 1-4). To determine the origin of these four RNA species, we eluted the RNAs from the gel and performed linker ligation, cDNA synthesis, and PCR amplification of each eluted RNA (see Methods). Sequence analysis of cloned PCR fragments identified the RNAs, although in all cases we also found contaminating rRNA and/or tRNA sequences.

Ten clones corresponding to RNA #4, the largest RNA, were sequenced and seven of these identified the RNA as the transfer messenger RNA (tmRNA; FTL_R001). This RNA is also designated SsrA and is 421 nt in size. tmRNAs are found in all bacteria and are highly expressed. The tmRNA has properties of both a tRNA and a mRNA. It participates in *trans*-translation, which is a reaction that transfers the translational complex to the tmRNA and ultimately leads to degradation of the polypeptide (for review, see [32]).

Based on the size of RNA #2, we presumed it was the 5S rRNA (114 nt). This was confirmed after cDNA sequencing. Next, RNA #1 was identified as the 4.5S RNA component of the signal recognition particle (FTL_R0044; 108 nt). FTL_R0044 was found in one of ten clones sequenced, whereas the other nine clones were the 5S rRNA. This is not very surprising considering the very similar size of these two transcripts and the high expression of the 5S RNA. Despite several attempts, we did not obtain any cDNA clones of RNA #3, the RNA with an apparent size of ~200 nt.

### Cloning and identification of additional sRNAs

As our analysis of highly expressed RNA did not result in the identification of novel small RNA transcripts, we proceeded to construct a limited cDNA library of low molecular weight RNAs. For this, we used the same approach as described for the highly expressed sRNAs. Total RNA from bacteria grown under the different conditions described above, but depleted of ribosomal RNAs (16S and 23S rRNA), was separated on a 8% denaturing polyacrylamide gel and RNAs of approximately 100-200 nt were eluted from the gel and processed the same way as for the highly expressed RNAs. The gel was divided into smaller areas before eluting the RNAs and ten clones from each area (shown in Figure 1B) were sequenced in order to identify new sRNA species. By this method, we identified two different RNAs, one of which was identified twice from the same gel piece, whereas the remaining clones contained rRNA, tRNA or other contaminating sequences.

**Figure 2:**
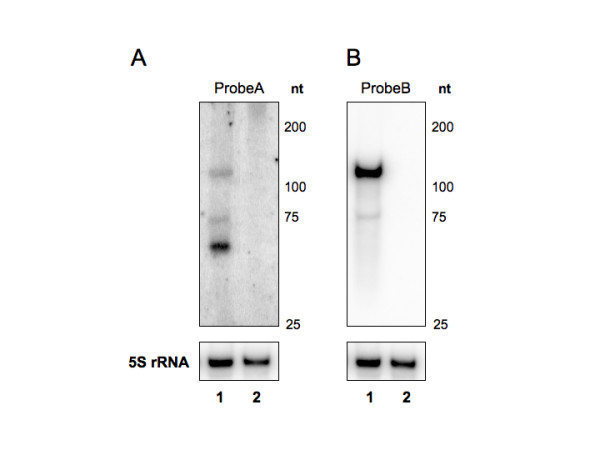
**Northern blots verify the presence of *F. tularensis *sRNAs**. RNA extracted from *F. tularensis *LVS (1) or mutant (2) bacteria in exponential phase was analyzed by Northern blotting using ^32^P-labeled oligonucleotides as probes (ProbeA specific for FtrA and ProbeB specific for FrtB). Approximate sizes are indicated based on RNA Marker and location of dyes in the gel.

To further confirm that the two identified candidates are authentic sRNAs, we performed Northern blotting analysis (Figure 2) using ^32^P-labeled oligonucleotides as probes. As can be seen in Figure 2, a strong band was observed for each of the sRNA candidates with sizes of approximately ~70 nt and ~110 nt, respectively. We refer to these sRNAs as FtrA and FtrB for *Francisella tularensis *sRNA A and B. In addition to the major bands, two bands of larger size were seen for FtrA and one with a lower size for FtrB, suggesting that processing of the transcripts might be occurring.

### sRNA mapping, localization, and structure analysis

To map the ends of the RNAs we performed 5'- and 3'-RACE (Table 1). This revealed that FtrA is 111 nt and FtrB is 115 nt in length. The size found for FtrB is in agreement with the strong bands seen in the Northern blot, whereas it seems to correspond to one of the larger, but less intense, bands seen in the Northern blot of FtrA.

**Table 1:** Small RNAs identified by cDNA cloning

sRNA	5' end^a^	3' end^b^	Length (nt)	Flanking genes	Genomic context^c^
FtrA	1256577	1256687	111	FTL_1319-FTL_1320	> > <
FtrB	37655	37769	115	FTL_0035-FTL_0036	> > >

^a ^Coordinate of the 5' end of sRNA (determined by 5'-RACE).

^b ^Coordinate of the 3' end of sRNA (determined by 3'-RACE).

^c ^The orientation of the sRNA and its flanking genes (in order of gene numbers). ">" designates a gene encoded on the coding strand and "<" designates a gene encoded on the non-coding strand.

**Figure 3:**
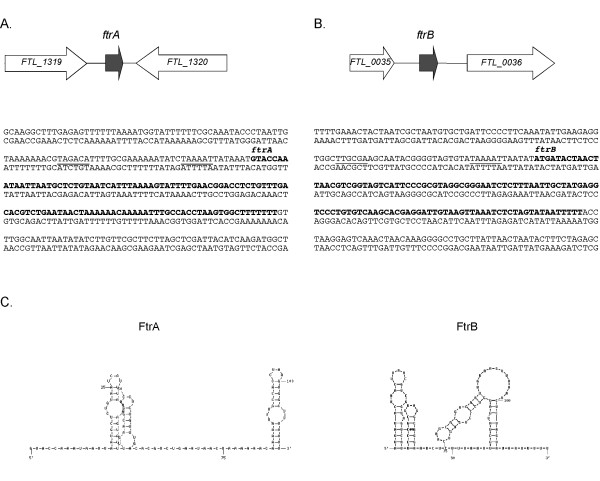
**Characterization of the two *F. tularensis *sRNAs**. Schematic representations of the genomic positions of the *ftrA *(A) and *ftrB *(B) loci are shown at top in each section. The DNA sequence of the regions, with the sRNA gene indicated in bold and proposed -10 and -35 boxes underlined, are shown below. The secondary structure predictions of FtrA and FtrB using Quikfold (RNA 3.0) are shown in (C).

The *ftrA *gene is located between *FTL_1319 *and *FTL_1320 *(Table 1 and Figure 3) and is encoded on the coding strand. The flanking genes encode a transposase and a conserved hypothetical protein. FtrB is encoded on the coding strand between *FTL_0035 *and *FTL_0036*, encoding two hypothetical proteins. The predicted secondary structures of FtrA and FtrB, using the Quikfold program http://dinamelt.bioinfo.rpi.edu/quikfold.php[33,34], are shown in Figure 3. Both FtrA and FtrB are predicted to be highly structured and contain several stem-loops.

To determine whether FtrA and FtrB are similar to known bacterial sRNAs, we performed BLAST to search the sequences of the non-coding RNA database http://ncrnadb.trna.ibch.poznan.pl/blast.html. No bacterial RNAs in the database showed sequence similarity to the two *F. tularensis *sRNAs. Sequences were also queried against the Rfam database http://rfam.sanger.ac.uk/, which did not result in any matches. Furthermore, a BLASTN search of the genes encoding either sRNA against the NCBI total sequence database showed that the genes are only found in *Francisella *and not in any other bacterial genome. This strongly indicates that the two RNAs are novel bacterial sRNAs.

### Characterization of *ftrA *or *ftrB *mutants

To study the role of the sRNAs, we proceeded by creating mutant strains carrying a chromosomal deletion of either the *ftrA *or *ftrB *gene. Sequencing and Northern blotting were respectively used to confirm deletion of these loci and the absence of their corresponding transcripts (Figure 2). Neither mutant strain exhibited any growth defects or increased sensitivity to several stress conditions (H_2_O_2_, ethanol, low pH, NaCl; data not shown), showing that deleting either *ftrA *or *ftrB *does not affect the ability of *F. tularensis *to survive a number of stresses. Likewise, we did not observe any change in expression of either FtrA or FtrB after exposure to oxidative or osmolarity stress (data not shown).

**Figure 4:**
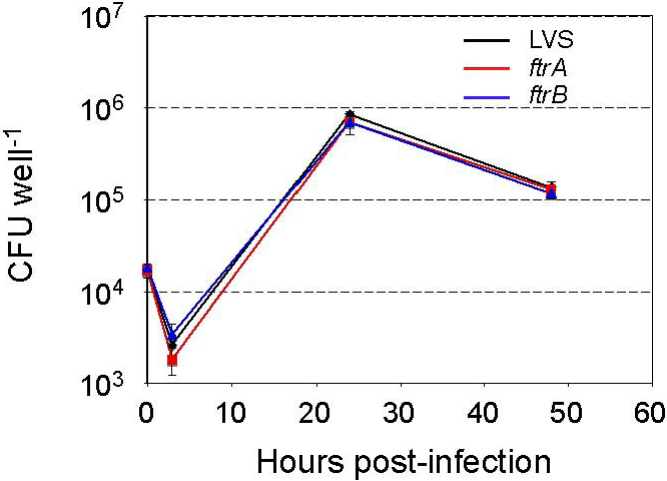
**Intracellular macrophage multiplication of sRNA mutants**. The number of intracellular bacteria was followed for 48 hours after infection of murine macrophage-like cells J774 by *F. tularensis *LVS wild-type or sRNA mutants *ftrA *or *ftrB*.

We next tested if either gene plays a role in *F. tularensis *intracellular multiplication. As shown in Figure 4, both mutant strains multiplied intracellularly in J774 murine macrophage-like cells in a manner indistinguishable from the wild-type strain, indicating that neither FtrA nor FtrB is required for multiplication in macrophages *in vitro*.

To examine if FtrA or FtrB is required for virulence of *F. tularensis*, we assessed the ability of each mutant strain to induce disease in the mouse model of infection. No major difference in survival was observed between mice infected with wild-type LVS or with either of the mutant strains, showing that neither gene is required for virulence (data not shown). In addition, after infecting mice with a 1:1 mixture of mutant and wild-type bacteria we recovered equal numbers of wild-type and mutant bacteria from the spleen (data not shown). This further demonstrates that deleting *ftrA *or *frtB *does not affect *F. tularensis *pathogenicity in the mouse model.

It is important to recall that most sRNAs affect gene expression negatively. Deleting a specific sRNA gene and thus alleviating the repressive effect may not be of major consequence for the bacterium. Our results from *in vitro *characterization of the mutant strains therefore do not rule out the possibility that FtrA or FtrB may indeed control functions related to growth, stress resistance or virulence.

### Identification of potential targets

Most characterized sRNAs control expression of their target genes by base-pairing with a target mRNA. In addition to affecting translation, base-pairing between a sRNA and its target frequently leads to changed stability of both sRNA and mRNA. Therefore, to experimentally identify potential targets for regulation by FtrA and FtrB, we compared the transcriptomes of LVS wild-type bacteria grown in regular broth to that of either the *ftrA *or the *ftrB *mutant. This way we identified several potential targets for FtrA and FtrB (Table 2). None of the potential targets identified by microarray analysis are located in the same genomic region as the sRNAs, indicating that the sRNAs are *trans*-acting. The analysis identified four genes for which the transcript was found at a higher level in the *ftrA *mutant than in LVS. These are *FTL_0045 *(encoding orotidine 5'-phosphate decarboxylase, PyrF), *FTL_0207 *(encoding pyrrolidone carboxylate peptidase, Pcp), *FTL_0902 *(encoding an oxidoreductase), and *FTL_1922 *(encoding a YggT family protein). Interestingly, *pyrF *transcripts were also found at higher levels in a *hfq *mutant [30]. Thus, deleting either *hfq *or *ftrA *affects *pyrF *mRNA levels in the same manner, in agreement with a regulatory mechanism in which Hfq-mediated FtrA-*pyrF *mRNA base-pairing leads to degradation of the target mRNA. This, however, needs to be demonstrated experimentally. The mRNA levels of five genes were changed in the *ftrB *mutant compared to wild-type bacteria: two were found in higher levels (*FTL_0836 *and *FTL_1754*) and three at lower levels (*FTL_0324, FTL_0421 *and *FTL_1966*) in the *ftrB *mutant. Of these, *FTL_0421 *(encoding a lipoprotein) was also found to be down-regulated in the *F. tularensis hfq *mutant [30]. This finding could be explained by a mechanism in which RNA duplex formation leads to stabilization of the mRNA. To confirm the results obtained by microarray analysis, we chose two genes with changed expression in either mutant strain and performed quantitative RT-PCR analysis. For each mutant strain, this analysis confirmed the change in expression for both genes (see Additional File 1). Presently we do not know if FtrA and FtrB regulate these targets directly as it is also possible that the observed changes in mRNA level are due to indirect effects of deleting the sRNA.

**Table 2:** Effect of *ftrA *and *ftrB *deletion on the transcriptome of LVS

Locus^a^	Gene product	Fold-change in *ftrA*^b^	Fold change in *ftrB*^b^
FTL_0045	orotidine 5'-phosphate decarboxylase	1.8	NA
FTL_0207	pyrrolidone-carboxylate peptidase	2.6	NA
FTL_0902	oxidoreductase	1.8	NA
FTL_1922	YggT family protein	10.8	NA
FTL_0324	pseudogene	NA	0.7
FTL_0421	lipoprotein	NA	0.7
FTL_0836	hypothetical protein	NA	8.2
FTL_1754	hypothetical membrane protein	NA	1.5
FTL_1966	anthranilate synthase component I	NA	0.7

^a ^Genes included had ≥1.5 average fold-change in mRNA level (calculated for all spots) and for which each of the five spots for the gene on the microarray had fold-changes ≥1.2

^b ^Change in mRNA level in mutant strain compared to wild-type LVS

### Identification of putative sRNAs using bioinformatics analysis

While our cloning based screen proved effective in identifying previously unknown sRNAs, the relatively small number of clones sequenced meant that only abundant small transcripts could be found using this approach. In an effort to identify putative sRNAs in *F. tularensis *that may have been missed in our physical screen, we used the bioinformatic tool SIPHT [35] to conduct a genome-wide bioinfomatic screen for candidate sRNAs in *F. tularensis *LVS. SIPHT identifies candidate intergenic loci based on the co-localization of intergenic conservation and Rho-independent terminators. It then annotates these candidates for several features, including the similarity of their primary sequence and predicted secondary structure to intergenic sequences in other bacterial replicons, their proximity to the putative binding sites of various transcription factors, and their homology to previously confirmed or predicted sRNAs and *cis*-regulatory RNA elements.

A total of 24 candidate loci were identified by SIPHT, including three intergenic regions (IGRs) each containing two overlapping candidate loci, leading to a total of 21 IGRs (Table 3 and Additional File 2). Importantly, both the FtrA and FtrB sRNA were identified in the bioinformatic prediction (Table 3, candidate 19 (FtrA) and candidate 14 (FtrB)), suggesting that other candidate loci identified by SIPHT may represent real non-coding RNAs. One of these candidates was annotated by SIPHT as sharing sequence homology with SprB, a previously identified but uncharacterized sRNA in *Staphylococcus aureus *[31], while another was found in a similar genomic location based on its flanking genes to P26, an sRNA of unknown function in *Pseudomonas aeruginosa*. For fourteen of the loci identified, including FtrA and FtrB, conservation of secondary structure was predicted by QRNA [36]. Putative sigma-70 promoters were identified within 400 nucleotides upstream of the predicted terminators of 9 candidates, including two upstream of *ftrA*, one of which in a position consistent with the larger band seen by Northern analysis and the results of the 5'-RACE.

**Table 3:** Putative sRNAs predicted *in silico*^a^

RNA	Length (nt)	Coordinates	Flanking genes	Genomic context^b^	Potential ORFs^c^
1	39	599871-599910	FTL_0609-FTL_0610	>68|>|984<	-
2	91	865504-865595	FTL_0886-FTL_0887	<1|>|129>	1
3	168	1039023-1039191	FTL_1090-FTL_1091	<4|>|-32>	8, 12*
4	141	1567719-1567860	FTL_1636-FTL_1637	>557|>|488<	2*
5	41	1808414-1808455	FTL_1875-FTL_1876	<349|<|203<	2, 8*
6	89	1251973-1252062	FTL_1313-FTL_1314	>145|<|47<	-
7	181	1240899-1241080	FTL_1303-FTL_1304	>26|<|0<	41
8	125	765395-765520	FTL_0777-FTL_0778	>132|<|0>	2
9	87	508319-508406	FTL_0527-FTL_0528	>149|<|0>	-
10	225	508181-508406	FTL_0527-FTL_0528	>11|<|0>	52*, 32, 37
11	191	361680-361871	FTL_0391-FTL_0392	<476|<|331<	-
12	229	133934-134163	FTL_0131-FTL_0132	<141|<|29<	3
13	92	52224-52316	FTL_0050-FTL_0051	<54|<|0<	7*, 2
14	177	37528-37705	FTL_0035-FTL_0036	>1|>|209>	10*, 4, 24*, 2
15	122	359671-359793	FTL_0389-FTL_0390	>1|>|176<	19, 2, 2
16	390	511941-512331	FTL_0529-FTL_0530	>1|>|-32<	9*, 4*, 7, 41, 7, 16*
17	52	527019-527071	FTL_R0021-FTL_0544	<2|>|8<	-
18	36	1251854-1251890	FTL_1313-FTL_1314	>26|<|219<	-
19	196	1256490-1256686	FTL_1319-FTL_1320	>57|>|128<	8, 9, 13
20	213	1351294-1351507	FTL_1420-FTL_1421	>67|<|0<	11, 19, 24*
21	119	1681442-1681561	FTL_1744-FTL_1745	<33|<|0<	15*, 3*
22	292	1229654-1229946	FTL_R0033-FTL_1289	>2|>|462<	10, 13*, 2, 11
23	228	49421-49649	FTL_0046-FTL_0047	>80|<|49<	2*, 11, 12*, 20
24	240	765280-765520	FTL_0777-FTL_0778	>17|<|0>	12*, 2, 33*

^a ^sRNA candidates, 5' and 3' ends predicted using SIPHT [35].

^b ^The orientation of the sRNA and its flanking genes (in order of gene numbers). ">" designates a gene encoded on the coding strand and "<" designates a gene encoded on the non-coding strand. Numbers outside of the "|" characters denote the distance between the boundaries of the predicted locus and its flanking genes.

^c ^Potential ORFs are indicated by their length in amino acids. * denotes an ORF staring with an alternative start codon.

For sequence of each RNA see Additional File 2.

Neither tmRNA nor 4.5S RNA were identified by SIPHT. Further analysis revealed that these loci were missed because they were not associated with predicted Rho-independent terminators. This may reflect a true paucity of Rho-independent terminators in *F. tularensis *or limitations in the ability of the three terminator predicting algorithms used by SIPHT to identify terminators in an AT rich genomes such as that of *F. tularensis*. The fact that these two sRNAs were not identified by SIPHT suggests that other *F. tularensis *sRNAs were likely missed in our screen.

Several characteristics of *Francisella *likely hinder predictions of sRNAs based on the proximity of intergenic sequence conservation and Rho-independent terminators. First, no genome sequences for close relatives of *F. tularensis *are available for BLAST comparisons and the relatively low GC content (32%) of the *F. tularensis *genome leads to spurious hits in BLAST searches that are more likely due to low sequence complexity than to actual sequence conservation. Second, as described above, it is also likely that existing terminator prediction programs, most of which had been trained on *E. coli*, are less effective in identifying terminators in AT-rich species such as *Francisella*. Indeed, in a kingdom-wide search for putative sRNAs in over 500 different strains, all *Francisella *sp. were among the 20 strains with the lowest density of putative intergenic Rho-independent terminators. However, while these factors likely contribute to both the decreased sensitivity and specificity of our sRNA predictions, the fact that both FtrA and FtrB were identified in our *in silico *screen suggests that the predictive algorithm used by SIPHT is effective in identifying novel sRNAs even in species such as *Francisella *that are AT-rich and have relatively few sequenced relatives.

Recently, a number of small ORFs encoding small peptides (<50 amino acids), previously non-annotated, have been identified in other bacterial species [37,38]. We therefore examined whether the predicted sRNA loci in *F. tularensis *contain ORFs encoding small peptides (Table 3). A few loci could encode small peptides of 24-52 amino acids, but most loci contained none or only very short ORFs, further supporting the hypothesis that the majority of the loci encode putative RNAs.

## Conclusions

Here, we have confirmed the expression in *F. tularensis *of the well-known non-coding RNAs tmRNA and 4.5S RNA after direct purification and cloning of the cDNAs. Two additional sRNAs expressed by *F. tularensis *were identified from a cDNA library prepared from low molecular weight RNA. The two putative sRNAs (called FtrA and FtrB), which are encoded in intergenic regions, were confirmed to be genuine sRNAs by Northern blot analysis and the transcripts were characterized with respect to transcription endpoints. The sRNAs do not show sequence similarity to any known sRNAs and their DNA sequences are only found in *Francisella *species, indicating that these are novel sRNAs. Deletion of either the *ftrA *or *ftrB *gene had no effect on bacterial survival during normal growth or stress, and did not affect the capacity of *Francisella *to induce disease in mice. This suggests that these sRNAs affect functions that are not required under the conditions tested or that additional sRNAs with redundant functions may exist. Specifically, *in silico *analysis identified conserved sequence between FtrB and sRNA candidate #20 (Table 3) suggesting they may be functional paralogues. Subsequently, microarray analysis allowed us to identify a number of potential targets of FtrA and FtrB regulation. Further experiments will be needed to verify if these represent real targets. Finally, *in silico *prediction of putative sRNA loci identified a number of *Francisella *IGRs, including the two harboring *ftrA *and *ftrB*. Future work will determine which of these IGRs indeed encode authentic sRNAs.

## Methods

### Bacterial growth and plasmids

All strains and plasmids used in this study are listed in Table 4. *F. tularensis *LVS was grown in Schaedler broth supplemented with vitamin K3 (Biomérieux, France), on chocolate agar plates supplemented with IsoVitalex vitamin solution (Biomérieux, France), or in defined medium [39]. *Escherichia coli *was grown in LB. When needed, media was supplemented with kanamycin (50 μg ml^-1 ^for *E. coli *and 10 μg ml^-1 ^for *F. tularensis*). Oligonucleotides used are described in Table 5.

**Table 4:** Bacterial strains and plasmids

Strain or plasmid		Description	Source or reference
**Strain**			
*F. tularensis*			
	LVS	subsp. *holarctica*, live vaccine strain	A. Sjöstedt
	LVSΔ*ftrA*	LVS with deletion of *ftrA*	This study
	LVSΔ*ftrB*	LVS with deletion of *ftrB*	This study
			
*E. coli*			
	DH5α		Strain collection
			
**Plasmid**			
	pCR2.1-TOPO	PCR cloning vector, Km^R^, Amp^R^	Invitrogen
	pMP812	*sacB *suicide vector, Km^R^	[40]
	pMP-Δ*ftrA*	pMP812 containing ~2 kb fragment for deletion of *ftrA*	This study
	pMP-Δ*ftrB*	pMP812 containing ~2 kb fragment for deletion of *ftrB*	This study

**Table 5:** Oligonucleotides used in this study

Oligonucleotide	Sequence (5' to 3' direction)^a^	Use
3' adapter	P-^-^rUrUrUCGGGCCGCGGACTGTidT	cDNA cloning and RACE
5' adapter	GATATGCGCGAATTCCTGTAGAACGAACACTAGGGGrArArA	cDNA cloning and RACE
5'PRIMER	GTAGAACGAACACTAGGGGAAA	cDNA cloning and RACE
3'PRIMER	GACAGTCCGCGGCCCGAAA	cDNA cloning and RACE
3' adapter_B	P-rUrUrUCTATCCATGGACTGTidT	cDNA cloning tmRNA
5' adapter_B	GATATGCGCGAATTCCTGTAGAACGAACACTAGAAGrArArA	cDNA cloning tmRNA
5'PRIMER_B	GTAGAACGAACACTAGAAGAAA	cDNA cloning tmRNA
3'PRIMER_B	TACAGTCCATGGATAGAAA	cDNA cloning tmRNA
ftrA_GSP_5'RACE	GTTATTCAGACGTGTCAAACAGAG	5'-RACE *ftrA*
ftrA_GSP_3'RACE	GTACCAAATAATTAATGCTCTGTAATC	3'-RACE *ftrA*
ftrB_GSP_5'RACE	GAGATTCCCGCCTACGCGG	5'-RACE *ftrB*
ftrB_GSP_3'RACE	GATACTAACTTAACGTCGGTAGTC	3' RACE *ftrB*
ftrA_DelR	GCGCGGCCGCGTGGTAAAATCATCTAGGTTCTAGC	Deletion *ftrA*
ftrA_DelB	CATTTATAATTTTAGATATTTTTTCGC	Deletion *ftrA*
ftrA_DelO	GCGAAAAAATATCTAAAATTATAAATGGGCAATTAATATATCTTGTTCGCTTCTTAGC	Deletion *ftrA*
ftrA_DelT	GC*GTCGAC*GCAACTAAGAAAAGAATATTTAATAGCC	Deletion *ftrA*
ftrA_DelCheck1	CATATGTAGTGTACTTTATTTAAATAC	Verification of *ftrA *deletion
ftrA_DelCheck2	CCTAAGTTTCAGTTGCTGAATTATTTGG	Verification of *ftrA *deletion
ftrA_DelCheck3	GCCACTGAAGGCGGAAATCTCGC	Verification of *ftrA *deletion
ftrA_DelCheck4	CAGTTAAATATTATTAACATTAAGAAAC	Verification of *ftrA *deletion
ftrB_DelR	GCGCGGCCGCCTTTTAAGATTTGTATTCTTATTTGTTC	Deletion *ftrB*
ftrB_DelB	CACTACCCCGTATTGCTTCGCAAGCC	Deletion *ftrB*
ftrB_DelO	GGCTTGCGAAGCAATACGGGGTAGTGCCTAAGGAGTCAAACTAACAAAGGGGCCTGC	Deletion *ftrB*
ftrB_DelT	GC*GTCGAC*CAGAGCATTTATGATAGTTTGTTTTCC	Deletion *ftrB*
ftrB_DelCheck1	CTAAATCTAAGGAATGATAATTAACC	Verification of *ftrB *deletion
ftrB_DelCheck2	GGACAGGAATGGACAGCAGAAG	Verification of *ftrB *deletion
ftrB_DelCheck3	GTATATCCTATTTGAAAAGCTAATGGC	Verification of *ftrB *deletion
ftrB_DelCheck4	CACTATATGGATATGCTTATGAACAAGC	Verification of *ftrB *deletion
ProbeA	CAGACGTGTCAAACAGAGGTCCGTTCAAAATAC	Northern blot
ProbeB	GAGATTCCCGCCTACGCGGGAATGACTACCGACG	Northern blot
FTL_0044_F	GCTATATGTCCCAGGTGTAAGG	qRT-PCR
FTL_0044_R	GCTCTTTGGCTTTTTTAGGGGTC	qRT-PCR
FTL_0421_F	GGGCAACTGTAACAGTTAAGC	qRT-PCR
FTL_0421_R	CTTCTTTGTCATAAACTACATTAGC	qRT-PCR
FTL_0836_F	GTGGCTATTGATGACATACTCAAC	qRT-PCR
FTL_0836_R	GCTAAGCCTAGATAACTGATACC	qRT-PCR
FTL_1922_F	GGATTTGATTTTTCTCCAATTATTG	qRT-PCR
FTL_1922_R	CTGCGCAATAATGCTTTGTATG	qRT-PCR

^a ^Bases preceded by r designates a ribonucleotide whereas all other are deoxyribonucleotides. idT designates an inverted deoxythymidine. P designates a phosphorylated 5' end. *Not*I site is underlined. *Sal*I site is shown in italics.

### Bioinformatic prediction of sRNA loci

Candidate sRNAs were predicted using the SIPHT program, as described previously [35]. Thresholds for BLAST E value, RNAMotif score, FindTerm score, and TransTerm confidence were set to 5e-3, -6, -10, and 86, respectively. All other parameters were set to default values.

### Extraction of RNA for gel fractionation

LVS was grown to mid-exponential phase or to stationary phase in Schaedler-K3 before harvesting the bacteria. For oxidative stress experiments, bacteria at OD_600 _= 0.3 were exposed to 10 mM H_2_O_2 _for 30 min before harvested. For experiments at high osmolarity, LVS was grown in complex medium containing 2% NaCl to OD_600 _= 0.3 before being harvested. Total RNA was extracted using Trizol reagent (Invitrogen) according to the protocol provided by the manufacturer and treated with Turbo DNase (Ambion). For cDNA library construction, total RNA was depleted of 16S and 23S rRNAs using the MICROB*Express *kit (Ambion) according to the protocol provided by the manufacturer before loaded on gel.

### RNA elution and cDNA cloning

20 μg of total RNA was heated and loaded onto an 8% polyacrylamide/8M urea gel with the RNA Century Plus (Ambion) as size marker. After separation, the gel was stained with ethidium bromide and a gel piece containing RNA of a certain size was cut out. RNA was eluted from the gel after crushing and incubation at 37°C in 0.5 M ammonium acetate and 1 mM EDTA (pH 8.0) for 6 hours under constant agitation. Supernatants were extracted once with chloroform before precipitation of the RNA with isopropanol in the presence of glycogen. Next, 3' adapter was ligated to the dissolved RNA with T4 RNA ligase in the presence of DMSO and RNase inhibitor for 90 min at 37°C. The RNA was purified from a 15% polyacrylamide/urea gel as described above to remove non-incorporated adapters. The eluted RNA was treated with tobacco acid phosphatase (TAP) before ligation of 5' adapter (using the same method as for 3' adapters) and subsequently non-incorporated adapters removed by passing and twice washing the RNA on a Microcon YM-30 (Millipore) column. The RNA was reverse transcribed using the 3'PRIMER and SuperScript II reverse transcriptase (Invitrogen), PCR amplified using 5'PRIMER and 3'PRIMER and ligated into pCR2.1-TOPO. All RNAs were identified using this protocol, except the tmRNA for which we used a protocol that ligates the 3'adapter before the 5'adapter and utilizes different adapters (adapters B; see Table 5).

### Northern blot

Total RNA was extracted using Trizol according to the manufacturer's instructions (Invitrogen) and quantified on a NanoDrop ND-1000 Spectrophotometer (Thermo Scientific). After extraction, 10 μg of total RNA were loaded on a polyacrylamide gel (8% acrylamide/8M urea). After migration, the RNA was transferred to a Hybond-N+ membrane (Amersham) and crosslinked with UV light. The membrane was prehybridized in Rapid-Hyb Buffer (Amersham). Then, ^32^P-labelled gene-specific probe (oligonucleotide, see Table 5) was added directly to the prehybridization buffer with the membrane and incubated for 16 hours at 42°C. After hybridization, the membrane was washed twice with 2×SSC/0.1% SDS, once with 1×SSC/0.1% SDS and twice with 0.1×SSC/0.1% SDS. Results were analyzed on a Storm 860 PhosphorImager (Molecular Dynamics) using the ImageQuant software (Molecular Dynamics).

### 5'- and 3'-RACE

For 5'-RACE, 15 μg of total RNA was incubated with TAP for 2 hours at 37°C after which 5' adapter was ligated with RNA ligase. RNA was purified from gel and reverse transcribed with a gene specific primer and SuperScript II RT. cDNA was then used as a template in PCR reaction with the 5'PRIMER and the gene specific primer (GSP_5'RACE) and cloned into pCR2.1-TOPO before sequencing.

3'-RACE was performed by ligating the 3' adapter to total RNA, followed by gel purification of adapter ligated RNA, reverse transcription with 3'PRIMER and finally conducting PCR with 3'PRIMER and GSP_3'RACE. The 3' end of each sRNA was determined by sequencing of cloned PCR products in pCR2.1-TOPO.

### Construction of *ftrA *and *ftrB *mutants

Regions of approximately 1 kb upstream and downstream of *ftrA *and *ftrB *were amplified by PCR using primer pairs DelR/DelB and DelO/DelT. The upstream and downstream fragments were purified from gel, annealed and extended in 20 cycles of PCR without primers and the product further used as template in a PCR reaction with primers DelR and DelT. The ~2 kb PCR products were cloned into pCR2.1-TOPO (Invitrogen) and mobilized as a *Not*I-*Sac*I fragment into the *sacB *based suicide vector pMP812 [40] creating pMP-Δ*ftrA *and pMP-Δ*ftrB*. pMP-Δ*ftrA *and pMP-Δ*ftrB *were introduced in LVS by electroporation and integration of the plasmid into the chromosome confirmed by PCR. Strains were then passed once in medium without selection, subsequently streaked on solid medium containing 6% sucrose and isolated colonies were tested for loss of the gene by PCR (using primer pairs DelCheck1/DelCheck4 and DelCheck2/DelCheck3). Deletion of the gene was confirmed by sequencing.

### cDNA labeling and microarray hybridizations

RNA used in microarray experiments was extracted using Trizol reagent combined with purification of the aqueous phase on RNeasy columns (Qiagen). cDNA labeling and microarray hybridizations were performed as described [30]. Three independent experiments and RNA extractions were performed and each set of RNAs was used in one hybridization experiment. The *F. tularensis *microarrays (obtained from the "Pathogen Functional Genomics Resource Center", PFGRC) contain 70-mer oligonucleotides, in five copies, representing all genes of strains SchuS4 and LVS. Microarrays were scanned with a Genepix 4000B scanner (Molecular Devices). To quantify signal fluorescence intensities, TIFF images were analyzed using the Genepix Pro 6.0 software. Statistical analyses were performed using publicly available software, the R/Bioconductor package LIMMA (available from http://www.bioconductor.org). A list of statistically significant differentially expressed genes was obtained using lowess normalization (after inspection of MA plots) and applying the empirical Bayes moderated t-test. Microarray data are available at ArrayExpress.

### Quantitative real-time RT-PCR

To validate the microarray results, two ORFs were selected (for each mutant strain) for real-time quantitative reverse transcription PCR (qRT-PCR) analysis with the same RNA samples used in the microarray hybridizations. For the analyses, one microgram of RNA was reverse transcribed using random hexamers and SuperScript II reverse transcriptase (Invitrogen) according to the protocol provided by the manufacturer. Real-time PCR was performed with gene-specific primers using an ABI PRISM 7700 and FastStart SYBR master mix (Roche Diagnostics). To calculate the amount of gene-specific transcript, a standard curve was plotted for each primer set using a series of diluted genomic DNA from LVS. To compare the transcript amounts in the different strains, the amounts of each gene transcript was normalized to DNA helicase (FTL_1656) as this gene has been shown to change little in expression during growth [23]. The expression of each gene was determined from three replicates in a single qRT-PCR experiment.

### Cell infections

J774 cells were propagated in RPMI or Dulbecco's Modified Eagle's Medium (DMEM) medium containing 10% fetal calf serum. Cells were seeded at a concentration of ~2 × 10^5 ^cells per well in 12-well tissue plates (Falcon) and monolayers were used 24 hours after seeding. J774 macrophage monolayers were incubated for 90 min at 37°C with the bacterial suspensions (approximate multiplicities of infection 100) to allow the bacteria to enter. After washing (time zero of the kinetic analysis), the cells were incubated in fresh culture medium containing gentamicin (10 μg ml^-1^) to kill extracellular bacteria. At several time-points, cells were washed three times in PBS and processed for counting of surviving intracellular bacteria. For this, bacteria were recovered by lysis of macrophages with distilled water and the titer of viable bacteria released from the cells was determined by spreading preparations on agar plates. For each strain and time in an experiment, the assay was performed in triplicate. Each experiment was independently repeated two times and the data presented are from one experiment.

### Mice infections

LVS and mutant strains were grown in Schaedler-K3 to exponential growth phase and diluted to the appropriate concentrations. 6 to 8-week-old female BALB/c mice (Janvier, Le Genest St Isle, France) were i.p. inoculated with 200 μl of bacterial suspension. Groups of five mice were inoculated with various doses of bacteria (approximately 10^1 ^to 10^4 ^bacteria) and the mortality was followed for 9 days. The actual number of viable bacteria in the inoculum was determined by plating dilutions of the bacterial suspension on chocolate plates. For competitive infections, LVS and mutant bacteria were mixed in 1:1 ratio and a total of approximately 400 bacteria were used for infection of five mice. After four days, mice were sacrificed. Homogenized spleen tissue from the five mice in one experiment were mixed, diluted and spread onto chocolate agar plates. PCR to distinguish wild-type and mutant bacteria were performed on 100 colonies. Animal experiments were performed according to the INSERM guidelines for laboratory animals' husbandry.

## Authors' contributions

GP carried out RNA extraction, cDNA library construction, Northern hybridizations, RACE experiments, qRT-PCR, mutant constructions and mutant characterizations. EF performed microarray hybridizations and analysis. JL carried out the bioinformatic sRNA prediction and annotations. MD carried out cell culture studies and ID the animal experiments. AC participated in design of study and writing of manuscript. KLM coordinated research, analyzed data, and wrote manuscript. All authors read and approved the final manuscript.

## Supplementary Material

Additional file 1 **Quantitative RT-PCR confirms the microarray results**. Transcript levels of selected genes were normalized to that of DNA helicase (FTL_1656) and the fold difference (in mutant strain relative to wild-type strain) and standard deviations are shown for the FTL_0045 and FTL_1922 genes (*ftrA *mutant) and FTL_0421 and FTL_0836 genes (*ftrB *mutant). Click here for file

Additional file 2 **Putative sRNAs predicted *in silico***. Table contains data provided in Table 3 in the text and the sequences of each of the predicted RNAs. Click here for file
