# Supplementary figures and images for: Identification of small RNAs in Francisella tularensis
Source: BMC Genomics. 2010 Nov 10;11:625. doi: 10.1186/1471-2164-11-625 (PMC3091763; doi:10.1186/1471-2164-11-625)

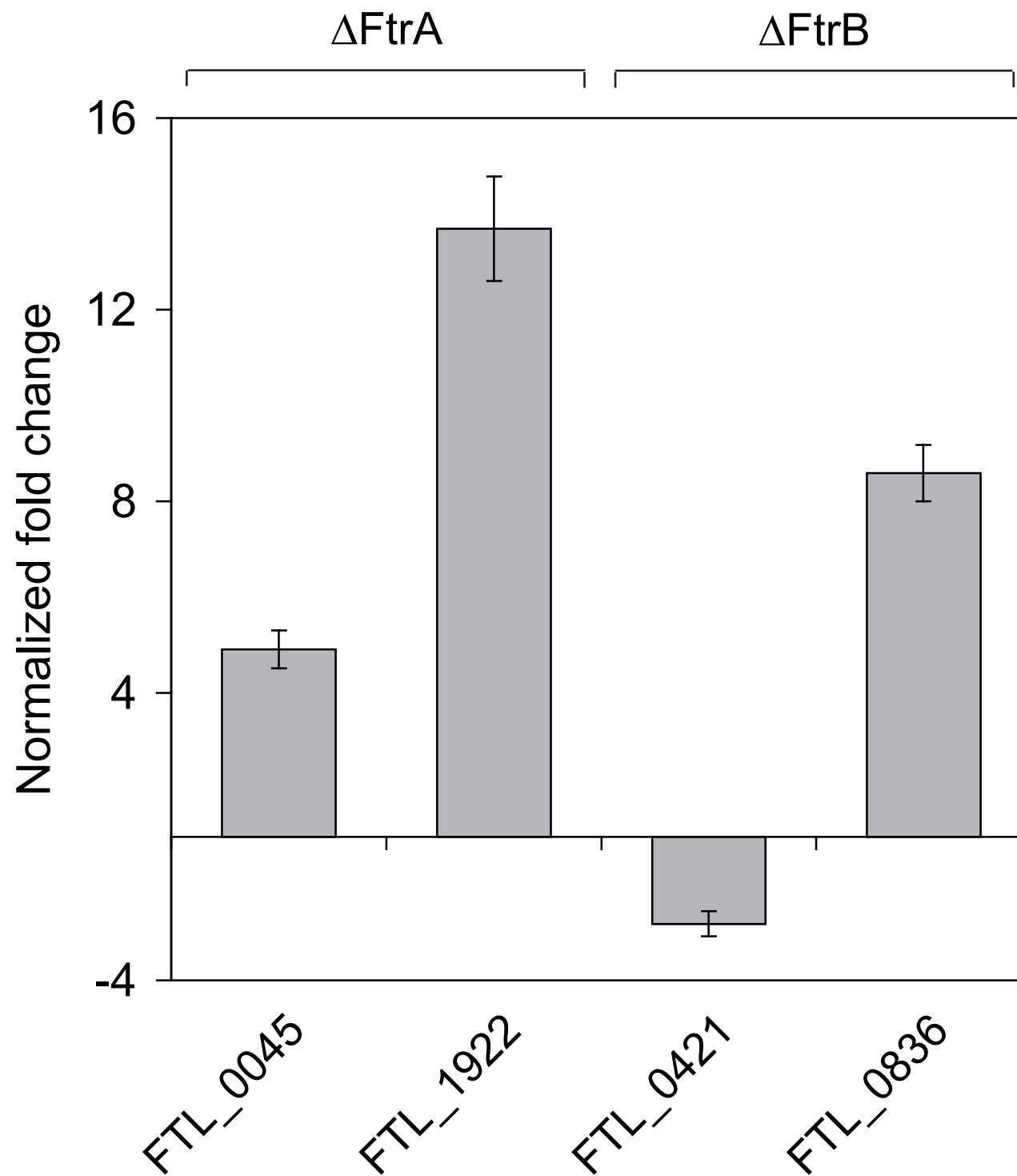

Supplement: Additional file 1 — Quantitative RT-PCR confirms the microarray results. Transcript levels of selected genes were normalized to that of DNA helicase (FTL_1656) and the fold difference (in mutant strain relative to wild-type strain) and standard deviations are shown for the FTL_0045 and FTL_1922 genes (ftrA mutant) and FTL_0421 and FTL_0836 genes (ftrB mutant). [file 1471-2164-11-625-S1.PDF]
