# Supplementary material for: Identification of small RNAs in Francisella tularensis
Source: BMC Genomics. 2010 Nov 10;11:625. doi: 10.1186/1471-2164-11-625 (PMC3091763; doi:10.1186/1471-2164-11-625)
Supplement: Additional file 2 — Putative sRNAs predicted in silico. Table contains data provided in Table 3 in the text and the sequences of each of the predicted RNAs. [file 1471-2164-11-625-S2.DOC]

| **RNA** | **Length (nt)** | **Coordinates** | **Flanking genes** | **Genomic context^b^** | **Potential ORFs^c^** | **Sequence (5’-3’)^d^** |
| --- | --- | --- | --- | --- | --- | --- |
| 1 | 39 | 599871-599910 | FTL_0609-FTL_0610 | >68|>|984< | - | AGCCACAGATAAAAATGTCATGCTGGATTTATTTCAGCG |
| 2 | 91 | 865504-865595 | FTL_0886-FTL_0887 | <1|>|129> | 1 | TAATCGAAACCCTTTATTTTTGTAGTATAGCTAAAGCGCTATTAATTAATGTAAAAATACACTTAACCGACCTGTGCTAATCTTGGCACGA |
| 3 | 168 | 1039023-1039191 | FTL_1090-FTL_1091 | <4|>|-32> | 8, 12* | AATTAATTAGCTAAGAATTTTGTTAGTTATTGTATGTTTTTTGGCGAGGTGTTTAAATAGCTTTACTGTGTATTTTTGCTATAGTTTGTCAGAAATGTT  AAGGTAAATATATATACTAAGAAATTTTTAGAGTAATTTTATGCAAAGAAAAAAAGGAGCTTATGCTCC |
| 4 | 141 | 1567719-1567860 | FTL_1636-FTL_1637 | >557|>|488< | 2* | TTGTTCATAAAATCTGATATAAATCATTTTTTTAAACTGAATAGATTCAACTATTTTATATACTGATTTATTATCAAAAGAGATTTTAGGAATAATAGGACTATAATAGCTGTATTAGTAACTTTTATTGGTGGCATAAGT |
| 5 | 41 | 1808414-1808455 | FTL_1875-FTL_1876 | <349|<|203< | 2, 8* | ATAGCCAAAGCGTGCATGGGATAGGCTTATGCGATTAGTAT |
| 6 | 89 | 1251973-1252062 | FTL_1313-FTL_1314 | >145|<|47< | - | CATATATACTCAACAACTACCGCAATACCCAACTTGATTGGGTATCTGACAAGTATTTGAGAGATTCCCACCTTCGTGGGAAATTCGTT |
| 7 | 181 | 1240899-1241080 | FTL_1303-FTL_1304 | >26|<|0< | 41 | TGTAATGCTGGTAAAAAATTTTACTATAACACCTCATTTAAATATAATCTATCATAAAAATCTCATACTTAATTTGTTTACTGAGTCAGTCAAAACAAA  GTCTTTTAAGATGACTAATATTAATTTCTAAGTTTGAAATAAAAAAAAGATACAAAAAAAACCAGCACAAGGCTGGTTTAGT |
| 8 | 125 | 765395-765520 | FTL_0777-FTL_0778 | >132|<|0> | 2 | AAACCAACCCCTACAATATTTTTTCACATATTTATAATTTGTCGTAGGGGCAATCCCTTGTGGTTGCCCTTGTATCCGTTATTTTAATTCAATTTATCGTAGGGGCTAACCTATGTGTTAGCCCT |
| 9 | 87 | 508319-508406 | FTL_0527-FTL_0528 | >149|<|0> | - | CAACATCTCTCTTATAGCTACCTGCATTATGCAACTTTTGCATAATGCTTTTTATTTGTGTGGTGTGTGTATTTTTTGCACATACCT |
| 10 | 225 | 508181-508406 | FTL_0527-FTL_0528 | >11|<|0> | 52*, 32, 37 | CAACATCTCTCTTATAGCTACCTGCATTATGCAACTTTTGCATAATGCTTTTTATTTGTGTGGTGTGTGTATTTTTTGCACATACCTATTTTATACTTCCAACTTGTGCATTTTTTACACTACTTAAATTTTCTATTCCTAGTAGTTTCAACTTGTAAGAATTATTTACAAGCTCACTTTTTAATAAACATCAATTTTAGTCATTCTACGGCTTGACCGTAGAAT |
| 11 | 191 | 361680-361871 | FTL_0391-FTL_0392 | <476|<|331< | - | TTTAAAGTACTACCCCGACCTGCTTCGCAATCTATCCAAAACCACCTCCCCTTGCAAGCAAGGTATTTCTCCAAAGGAGGAGAATAATAACTTATATTTAGCTAGTACCAAATTCCCCTTCAAAATTTTGAAGGAGTGGCAGGCGCTAGACACCACACTCTACCTATCGGCACCCCTATTAAGAGGGGAAT |
| 12 | 229 | 133934-134163 | FTL_0131-FTL_0132 | <141|<|29< | 3 | TAAAAAAATCATTAATACGCTATAATCTAGGTTTACACTAAAATATTATATAGCTACAATAACTATATCTATTTTAAATAATGATATTTTAGTCAAAATATTATATAAACAAAAAGATAAGGGAAAATGCATAAAAAAGTAGGTTCACGTATACAGATACGAAGACATCGAGAAGATAATTTCTCCACTGTCGAAACATTTAGTAGTAGTGGTGATTCAGCTATTGCTA |
| 13 | 92 | 52224-52316 | FTL_0050-FTL_0051 | <54|<|0< | 7*, 2 | CAATACATACCGCATATAGCAAGAGTGGCAAGAGTGAGTCGCGATTAAATGTTTTAAACTTTTTCTTTGGTGCGGGCTCTGCTTTTGTACCT |
| 14 | 177 | 37528-37705 | FTL_0035-FTL_0036 | >1|>|209> | 10*, 4, 24*, 2 | AGAATTTAATCTTTGTTTTGCTAATCATTTGTTTTTGAAACTACTAATCGCTAATGTGCTGATTCCCCTTCAAATATTGAAGAGGTGGCTTGCGAAGCAATACGGGGTAGTGTATAAAATTAATATATGATACTAACTTAACGTCGGTAGTCATTCCCGCGTAGGCGGGAATCTCTT |
| 15 | 122 | 359671-359793 | FTL_0389-FTL_0390 | >1|>|176< | 19, 2, 2 | TGAATTATGTTGTATGGGCTAACCAATGTGTTAGCCCTTGTGGTATTGGTGCTAAAGAAGGGTTGACACATGGTTCAACCCCTACAAAAACTGTTGTCATTCCCACGTAGGTGGGAATCTTT |
| 16 | 390 | 511941-512331 | FTL_0529-FTL_0530 | >1|>|-32< | 9*, 4*, 7, 41, 7, 16* | CGATTCCATTATGTGCAGCTTAGAAAAAAAATAATTACTTAACAATATTCCAAGGATCTTGATATTGAGTATGGTTTTCTACAGGGTTAACTCCTTTATTCCACCATTGTGCTGCGTATTTGACACCATTGATTTCGACAGTGTCGCCAGCAACATACTCTTTTGTAGGGTCCCATCTGGAGTTGGTTTAGGTTGAGGATTTAAATTTTCCCAGGCAGCATCAGAGAAGTTGCCTAATGGAGTATAGGCAGGAGATTGTTACACCAGTTAGCCTCTTTACATTTATACTCACTATCACCTGCTTTCACTACAGTTCCATTTACATAGCTACCGATACCATCCGGATATGTATATTGAGGTTATGGAGTAGGTCCAGGCTCTGGACTTGGT |
| 17 | 52 | 527019-527071 | FTL_R0021-FTL_0544 | <2|>|8< | - | AACTTAGAGACGATTATATTTTAAATAGAACTGTTAGTCAAGGACTATTTTT |
| 18 | 36 | 1251854-1251890 | FTL_1313-FTL_1314 | >26|<|219< | - | GGATTATTCTCCTCCTATGGAGGAGTACCTTGCTTG |
| 19 | 196 | 1256490-1256686 | FTL_1319-FTL_1320 | >57|>|128< | 8, 9, 13 | TTTGAGAGTTTTTTAAAATGGTATTTTTTCGCAAATACCCTAATTGTAAAAAAACGTAGACATTTTGCGAAAAAATATCTAAAATTATAAATGTACCAAATAATTAATGCTCTGTAATCATTTAAAAGTATTTTGAACGGACCTCTGTTTGACACGTCTGAATAACTAAAAAACAAAAATTTGCCACCTAAGTGGC |
| 20 | 213 | 1351294-1351507 | FTL_1420-FTL_1421 | >67|<|0< | 11, 19, 24* | CCATGATTCTAGAGATTCCTTCCTACAAGAGAATTACAAAAATGTATAGTCACACCTATTAAGTCGGGTATCTCTCCAGTATTTAAGAGAGCCCCGTGTCAAGCACGAGGATGACTGCTATTTTTTTATGGTACATACTGATTATATCAACAGTCTTTTGTAGCCTATAACTCTGCTGTGATACCCGCGAAGGTGGGTATCTTTAGGATAGTG |
| 21 | 119 | 1681442-1681561 | FTL_1744-FTL_1745 | <33|<|0< | 15*, 3* | CTAAACTATAGTTTTTAGAAATTTATGACTAGTGGTCAATTTTTGGTCCCTAGTCTTTCTAGTATTGAAAGATACATAGATATTGGTGCAAATATAATCATATTTGCTTTTTATTTTGT |
| 22 | 292 | 1229654-1229946 | FTL_R0033-FTL_1289 | >2|>|462< | 10, 13*, 2, 11 | GTAATTTCAATGCTTTCAGCCCTACACATCAAAACACTATAAATTATCAAGGTGTATATAAGGATCACTAGGTCTAATTTTTTAAAGATATAAACCTGCTCAATACAATCTACTGAAATAAACAATTTCTTTTCAAAACAAACAAATGTTTTGAAAGTATTATCTGAAACGCTAGTTGATTTAGATAGCCCTGAGGCTATATAAATCTTTTACAATAGCGTTTCTAAGTATTAATCATGCGAAGCATTACGCCTTGCCCACCGTCGGCCTGATAAGGTTGACCAACGGGTTT |
| 23 | 228 | 49421-49649 | FTL_0046-FTL_0047 | >80|<|49< | 2*, 11, 12*, 20 | TATATCTTGAAGCTAGATTCTACGATCAAGTCGTAGAATGACATATGCCTTTCAAATTAGATATAGAAAGTAGTGCATTAGCAATTAGTAATTTCAAAAGTAAATAATTTGAAAATAAAACTGCTTGAACAAAGTGAGTTTTTTATTTCCTTTTTGCTTTGAACTACGTTAGCGTAGCACTAAGCAGTTTTGCATACAATTCCCCTCTTAAGAGGGGCGTCATTTTAA |
| 24 | 240 | 765280-765520 | FTL_0777-FTL_0778 | >17|<|0> | 12*, 2, 33* | AAACCAACCCCTACAATATTTTTTCACATATTTATAATTTGTCGTAGGGGCAATCCCTTGTGGTTGCCCTTGTATCCGTTATTTTAATTCAATTTATCGTAGGGGCTAACCTATGTGTTAGCCCTATTTTATATTATGGTTTATATATCATAACGGGTTGACACACAGGTCAACCGCTACCACACATTTTCACAAATCCATAGTTTATTGTAGGGGCAGATTCTATATCTGCCCTTTTAT |

^a^ sRNA candidates, 5’ and 3’ ends predicted using SIPHT [35].

^b^ The orientation of the sRNA and its flanking genes (in order of gene numbers). “>” designates a gene encoded on the coding strand and “<” designates a gene encoded on the non-coding strand. Numbers outside of the “|” characters denote the distance between the boundaries of the predicted locus and its flanking genes.

^c^ Potential ORF are indicated by their length in amino acids. An asterics indicates …

^d^ Sequence of predicted sRNA. For sRNAs encoded on the + strand this corresponds to the + strand and for sRNAs encoded on the – strand this correspond to the - strand.
